# A Versatile Approach to Stabilize Whispering Gallery Microresonators Toward Reliable Photonic Labeling

**DOI:** 10.1021/acs.jpclett.5c03840

**Published:** 2026-01-29

**Authors:** M. Reale, A. Madonia, S. Agnello, M. Cannas, E. Marino, A. Sciortino, F. Messina

**Affiliations:** † Department of Physics and Chemistry E. Segrè, 18998 University of Palermo, via Archirafi 36, 90123 Palermo, Italy; ‡ Consorzio Interuniversitario Nazionale per la Scienza e Tecnologia dei Materiali (INSTM), via G. Giusti 9, 50121 Firenze, Italy; § ATEN Center, University of Palermo, Viale delle Scienze Ed. 18, I-90128 Palermo, Italy

## Abstract

Whispering gallery mode (WGM) microresonators provide ultrasensitive optical fingerprints that are ideal for photonic labels capable of detecting minute variations in size and refractive index. WGM microresonators can be obtained through convenient self-assembly protocols, for instance, by loading polystyrene microparticles with colloidal quantum dots. However, despite their ultrasharp spectral signatures, these resonators exhibit significant thermal and temporal drifts that limit their applications. We combine experiments and modeling to show that these drifts originate from the slow, days-long release of residual solvent trapped within the microparticles, which can be monitored via WGM spectroscopy. We establish a simple protocol to suppress this instability, locking the resonances into stable, sharply defined modes. This approach provides a straightforward route for long-term stabilization of WGM microresonators, advancing their reliable use as robust optical platforms, noninvasive sensors, and precise self-trackers of physicochemical changes at the microscale.

## Graphical abstract

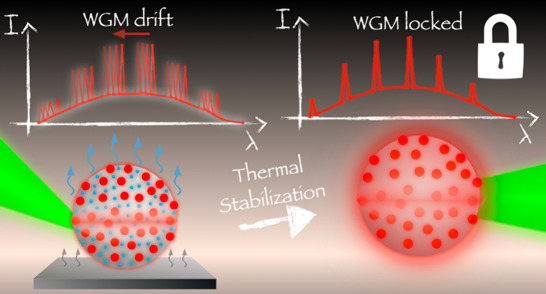

Optical microresonators exploit their symmetry to confine light near interfaces. As light circulates along a closed loop, constructive interference occurs when the optical path length matches an integer multiple of the circulating wavelength. When this condition is met, sharp resonances, known as Whispering Gallery Modes (WGMs), arise with ultrahigh quality factors *Q* = λ/Δλ and relatively small mode volumes.^1,2^ Together, these characteristics provide a platform of exceptional sensitivity for microscale optical sensing and spectroscopy. Minute variations in size or refractive index can produce measurable shifts, making WGMs ideal for applications ranging from biosensing^3,4^ and microlasing^5–7^ to optical labeling and anticounterfeiting technologies,^8–10^ where the dense resonance patterns serve as physically unclonable spectral fingerprint. Dielectric spheres, rings, toroids, and other more complex morphologies can serve as WGM microresonators.^1,2^ Despite their potential, practical use of WGM microresonators is often hindered by the complexity of light coupling into/from the resonator, requiring, for instance, carefully approaching a tapered fiber or prism to the surface of the microresonator. Active WGM microresonators have been developed to overcome these limitations by embedding a gain medium guest within the resonator host.^1^ In such systems, spontaneous emission couples directly to the cavity modes, eliminating the need for external optics and enabling a straightforward optical readout while preserving the intrinsic sensitivity of WGMs.

Colloidal semiconductor quantum dots (QDs) are particularly attractive as a gain medium due to their high photoluminescence (PL) quantum yield, tunable emission, and photostability.^11^ Polymeric microspheres, such as polystyrene microparticles (PSμPs), offer an ideal host, as they are optically transparent in the visible range, chemically inert, easy to functionalize, and can be prepared in various, well-controlled sizes.^12,13^ Coupling QDs to PSμPs via solvent-assisted loading is straightforward, providing a scalable strategy for the synthesis of active microresonators,^14–18^ with applications spanning microlasing,^7,15^ optical labeling,^19^ enhanced electron or energy-transfers,^18,20^ and environmental sensing.^14,16,17^

WGMs can act as ultrasensitive, noninvasive real-time probes of the resonator’s geometry or refractive index, thus enabling the monitoring of processes such as polymer relaxation,^21^ internal refractive index changes,^22^ and vapors or solvent uptake.^23–25^ However, the same exceptional sensitivity that makes WGMs such powerful probes also represents a well-recognized practical limitation: minute and often uncontrolled variations in the microresonator or its local environment can induce substantial spectral drifts, ultimately compromising specificity and long-term stability.^26–28^ Understanding the physicochemical origin of these drifts and how to suppress them is therefore essential for enabling reliable WGM-based technologies.

Here, we overcome this limitation by demonstrating an effective route to obtain drift-free active WGM microresonators. We use model hybrid microresonators in which colloidal QDs are coupled to PSμPs, providing a well-defined WGM fingerprint that allow us to identify slow solvent desorption as the dominant driver of irreversible blueshift of the resonances. Real-time spectral tracking under controlled environmental conditions directly links solvent loss to the optical response of the resonator. A thermal activation step fully suppresses these drifts, locking the WGM pattern into stable, low-thermosensitivity microresonators as robust and scalable platforms for secure photonic labeling and real-time monitoring of physicochemical changes on the nanoscale.

We synthesized CdSe/CdS core/shell QDs following the literature.^29^ As reported in Figure 1a, the QDs exhibit an absorption spectrum dominated by the CdS shell edge below 550 nm, while the CdSe core excitonic feature remains discernible at around ∼610 nm (Figure S1). Figure 1a also shows the PL spectrum, centered at ∼625 nm (PL Quantum Yield 60%), which is compared in Figure S1 with the excitonic structure resolved in the absorption spectrum. We integrated these emitters into PSμPs (10 μm diameter) by using a solvent-assisted self-assembly strategy adapted from the literature and illustrated in Figure 1b.^18^ Briefly, QDs dispersed in chloroform were added to a suspension of PSμPs in isopropanol while stirring. Chloroform is known to swell polystyrene^30^ which may facilitate limited QD diffusion into the outer polymer layer, while its partial miscibility with isopropanol promotes mild colloidal destabilization that favors QD deposition onto the PSμPs.^31,32^ After 12 h, QD-PSμPS were separated from unbound QDs via centrifugation and redispersed in isopropanol. Further details can be found in the Experimental Methods section.

**Figure 1:**
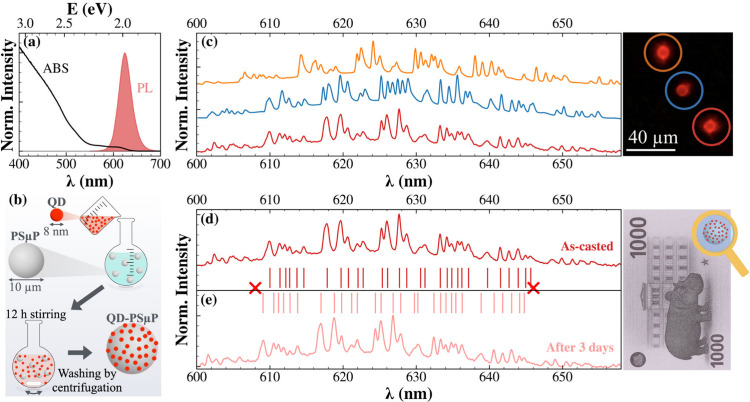
(a) Absorption (ABS) and photoluminescence (PL) spectra of a QD dispersion in chloroform. (b) Reaction scheme for QD incorporation into polystyrene microparticles (PSμPs), to obtain QD-PSμPs. (c) μ-PL spectra collected from distinct individual QD-PSμPs (vertically shifted), as imaged on the right of the panel. (d) Comparison between the μ-PL spectrum of the same QD-PSμP as-casted and (e) after 3 days under ambient conditions. Vertical red lines help to identify the individual modes appearing in the spectra. Although the well-resolved WGM structure makes these spectra ideally suited for photonic barcoding, the observed spectral drift currently hinders their reliable use as photonic microlabels (right).

After dropcasting, we observe that individual PSμPs emit bright red light consistent with the photoluminescence of the QDs, indicating that QDs have been deposited onto the microparticles. Interestingly, the resulting microphotoluminescence (μ-PL) spectra of individual QD-PSμP (Figure 1c) show a dense array of narrow WGM peaks superimposed on the broader QD emission envelope. μ-PL spectra from distinct QD-PSμPs exhibit discrete WGM progressions, producing unique spectral patterns as exemplified in Figure 1c. This spectral diversity reflects the extreme sensitivity of WGM resonances to the geometry and refractive index of the microresonator. The resonance wavelength *λ*_*l*__,_ *_i_* for a mode of angular mode number *l*, radial mode number *i*, and polarization *p*, which can be transverse electric (TE) or transverse magnetic (TM), is described by the asymptotic expansion derived from the Debye approximation of the Mie scattering solution:^1,33^

1 $$\lambda_{l,i}=2\pi Rn_{eff}{\left[\nu +\alpha_{i}{\left(\frac{\nu }{2}\right)}^{1/3}-\frac{p}{\sqrt{m^{2}-1}}+\frac{3\alpha_{i}^{2}{\left(4\nu \right)}^{-1/3}}{10}-\frac{p\left(m^{2}-2p^{2}/3\right)}{{\left(m^{2}-1\right)}^{3/2}}\frac{\alpha_{i}}{{\left(2\nu^{2}\right)}^{1/3}}+O\left(\nu^{-1}\right)\right]}^{-1}≔\frac{2\pi Rn_{eff}}{Q\left(\nu ,i;n_{eff},n_{env}\right)}$$

where $\nu =\frac{1}{2}+l$ , *R* is the sphere radius, *n*_*eff*_ is the effective refractive index of the sphere, *m* = *n*_*eff*_/*n*_*env*_ is the refractive index contrast between the sphere and the refractive index of the surrounding environment medium, *α*_*i*_ the *i*-th zero of the Airy function, and *p* = *m* for TE or *p* = 1/*m* ^2^ for TM modes. As shown in the simulations reported in Figure S2, 0.1% variations in size or refractive-index produce nanometric shifts in the resonance positions. Consequently, each microparticle exhibits a unique and irreproducible spectral “fingerprint”. This inherent spectral individuality offers a compelling route toward several applications, such as photonic microtagging and anticounterfeting labels. Each QD-PSμP can act as an optical microlabel whose μ-PL spectrum encodes an unclonable optical signature, as conceptually illustrated in Figure 1d-e. Indeed, the narrow WGM peaks function as a photonic barcode that cannot be replicated even under nominally identical fabrication conditions, thus enabling physically unclonable functions.^8,10,34^ Such optical microlabels could provide secure identifiers for anticounterfeiting or secure authentication technologies, where information is stored and verified through spectral readout. Potential applications extend to secure labeling of high-value goods, tracking of individual components in complex assemblies, or any scenario requiring the distinction of nominally identical micro-objects.

A crucial requirement for these applications is the temporal stability of the spectral barcode. To probe this stability, we tracked the μ-PL spectrum of individual QD-PSμPs over time. Surprisingly, the spectral fingerprints do not remain static. As shown in Figure 1e, the WGM pattern recorded 3 days after dropcasting under ambient laboratory conditions exhibits more than 1 nm blueshift compared to the as-cast spectrum (Figure 1d). This spectral drift can stem from subtle morphological or environmental changes—such as polymer relaxation, QD redistribution, or adsorption/desorption of solvent molecules—that alter the local refractive index and/or the effective cavity radius (see eq 1).^21,23,24^ Although this observation highlights the extraordinary sensitivity of WGMs to minute perturbations, it simultaneously challenges their use as reliable photonic labels, hinting that further control over the microresonator environment is needed.

We studied the spectral instability in different environments to identify its cause. In a first experiment, we monitored the evolution of μ-PL spectra of a QD-PSμP over time in the first few minutes after drop-casting, as schematically illustrated in Figure 2a. Immediately after drop-casting from isopropanol, the WGM structure is barely discernible on the PL envelope (Figure 2b), with broader peaks, which suggests a low Q-factor. This is likely due to the presence of solvent at the particle-environment interface, since the QD-PSμP is entirely exposed to liquid isopropanol as surrounding medium. Interestingly, the WGM spectrum displays several sets of two closely spaced resonances, each originating from a pair of a TE and a TM mode. The separation between two consecutive TE (or TM) modes is ∼7.5 nm, corresponding to the spacing between consecutive azimuthal mode numbers (Δ*l* = 1). Using the standard expression for the free-spectral range^1^ Δλ_Δ_ *_l_*_=1_ = λ^2^/2*πRn*_*eff*_, and assuming a 10 μm PSμP with *n*_*eff*_ = *n*_*PS*_ = 1.58 at λ = 630 nm, we obtain Δλ ≈ 7.9 nm, in good agreement with the experimental value. This indicates that under these initial conditions where the refractive index contrast between the polystyrene sphere and the surrounding medium is reduced by residual isopropanol, the spectrum is dominated by fundamental radial modes (*i* = 1). After 2 min, the peaks dramatically narrow, additional modes appear, consistent with the emergence of higher-order radial modes,^35^ and the progression of WGMs undergoes a blueshift. After five min, the spectrum appears to stabilize, corresponding to the evaporation of the isopropanol outside the QD-PSμP.

**Figure 2:**
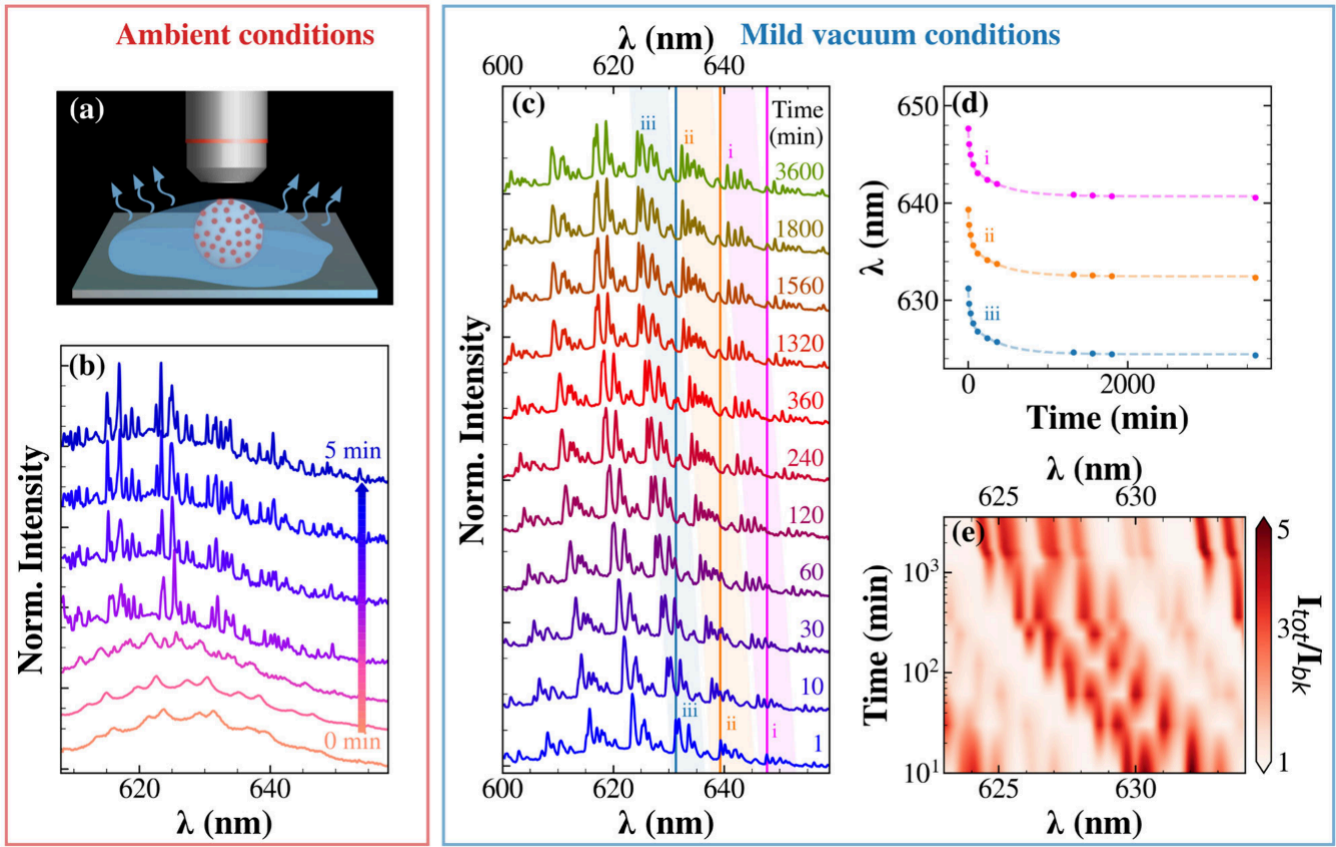
(a) Schematic illustration of the experimental geometry used to collect the PL spectra from a single QD-PSμP under ambient conditions during isopropanol evaporation. (b) Time evolution of the PL spectra acquired from the same QD-PSμP after drop-casting under ambient atmosphere. (c) Evolution of the PL spectra of an individual QD-PSμP stored under mild vacuum conditions (pressure: 0.15 bar) as a function of storage time. Colored vertical lines and shaded regions highlight the progressive blueshift of three representative peaks. (d) Temporal kinetics of the spectral positions of the three representative WGM peaks marked in panel (c). (e) Wavelength–time colormap of the ratio between the total emission intensity and the background PL intensity within a selected spectral window, emphasizing the spectral drift of the WGM peaks.

Then, we tested for the presence of residual solvent by placing a QD-PSμP under mild vacuum conditions (pressure 0.15 bar), measuring the spectrum at different intervals. Figure 2(c) shows a further progressive blue-shift of the WGM pattern. Interestingly, 10 min under reduced pressure induce a spectral shift exceeding the 1 nm shift observed after 3 days in air (compare with Figure 1), compatible with a sped-up isopropanol evaporation under reduced pressure. The temporal evolution of representative WGM peaks is plotted in Figure 2d. A biexponential fit yields mean time constants of 25 and 364 min, both associated with shift amplitudes of 3.4 nm and likely reflecting internal solvent release from regions with different effective diffusion lengths within the polymer matrix. The spectral shift of a specific set of resonances is visually highlighted in Figure 2e, which presents a colormap of the total PL intensity (normalized to the broadband PL) to illustrate how WGM features shift during vacuum exposure. As more clearly evidenced in Figure S3, after 3600 min under low pressure, not only does the WGM spectrum exhibit a ∼7 nm blue shift but the substructures of certain modes become more distinctly resolved.

We propose that a two-step mechanism occurs (Figure 3) that rationalizes the spectral behavior observed during the evaporation of isopropanol. Additional details can be found in the Supporting Information.

**Figure 3:**
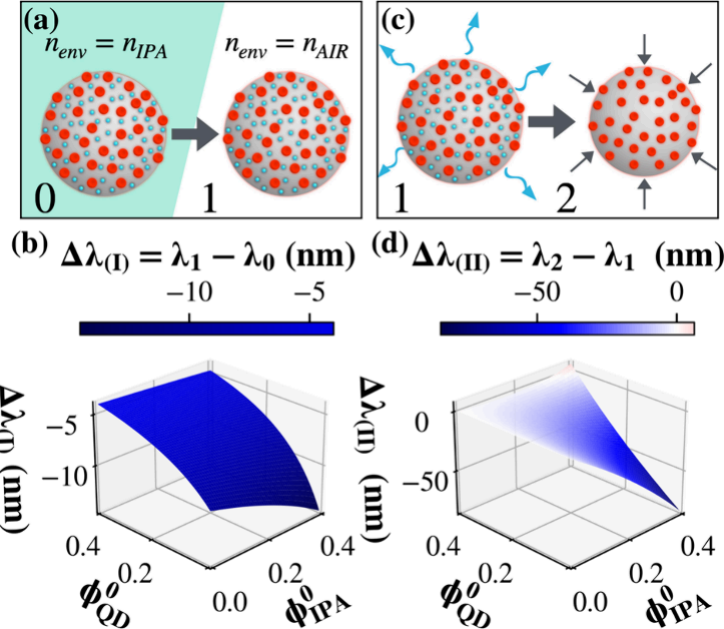
(a,c) Schematic representation of the two sequential stages of isopropanol evaporation, affecting the WGM spectral position in a PSμP: (a) transition from immersion in isopropanol (n_env_=n_IPA_) to air (n_env_=n_AIR_), leading to a shift from λ_0_ to λ_1_; (c) subsequent evaporation of the residual impregnating IPA, inducing particle shrinkage and a further shift from λ_1_ to λ_2_. (b,d) Simulated wavelength shifts Δλ_(i)_= λ_1_- λ_0_ and Δλ_(ii)_= λ_2_- λ_1_ as a function of the initial volume fractions of QDs (Φ^0^_QD_) and IPA (Φ^0^_IPA_) within the microparticle.

(i) *External Solvent Evaporation and Increase of Refractive Index Contrast*
  Immediately after drop-casting, the particle is immersed in isopropanol, so that the surrounding refractive index is ${n_{env}=n}_{IPA}$ (Figure 3a). As isopropanol evaporates, *n*_*env*_ progressively approaches *n*_*AIR*_, increasing the refractive index contrast and sharpening the WGM resonances by enhancing the cavity Q-factor. During this stage, the QD-PSμP retains a small volume fraction of isopropanol ϕ_0_ *^IPA^*. Similarly, if ϕ_0_ *^QD^* denotes the initial QD volume fraction, the effective refractive index of the hybrid particle can be estimated using the Maxwell–Garnett effective medium approximation, using the refractive indices of polystyrene (*n*_*PS*_), isopropanol (*n*_*IPA*_), and QDs (*n*_*QD*_). By applying eq 1, we simulate the spectral shift Δλ_(_ *_I_*_)_ expected when the external medium changes from *n*_*IPA*_ to *n*_*AIR*_, as a function of the initial volume fractions ϕ_0_ *^QD^* and ϕ_0_ *^IPA^*. We explored a wide and conservative range of initial volume fractions, up to 0.4, which significantly exceeds what is expected for a nonporous matrix. The resulting 3D map (Figure 3b) shows that, regardless of the specific values of these parameters, a net blue-shift of several nanometers is expected during initial evaporation of liquid isopropanol, in agreement with the experimental drift of the WGM peaks observed in Figure 2b.
(ii) *Internal Solvent Loss and Structural Shrinkage*
  Once the isopropanol outside the particle has evaporated, additional solvent trapped in the polymer matrix gradually desorbs. The progressive loss of this retained solvent can lead to a slight densification or contraction of the polymer network, producing a gradual decrease in the particle radius and a continuous reduction of isopropanol volume fraction from the initial ϕ_0_ *^IPA^* value to zero (Figure 3c). Using eq 1, we evaluate the additional spectral shift Δλ_(_ *_II_*_)_ associated with this internal solvent loss. As shown in Figure 3d, Δλ_(_ *_II_*_)_ is consistently negative over a wide range of realistic ϕ_0_ *^QD^* and ϕ_0_ *^IPA^*, again in agreement with the experimentally observed blueshift. Overall, the spectral evolution reflects a coupled optical-structural response of the hybrid microresonator: both refractive-index contrast and cavity size evolve as solvent is released, driving the characteristic two-step WGM drift.

We gain deeper insight into the solvent release process by monitoring the μ-PL spectrum *in situ* while gradually increasing the temperature of a QD-PSμP. For each temperature, a μ-PL spectrum was acquired after approximately 20 min of thermal equilibration. Temperatures above 90 °C were avoided since polystyrene exhibits a glass transition in the range 95–100 °C,^36^ beyond which polymer morphology and optical properties could irreversibly change.

In a first experiment, we measured a QD-PSμP stored for 1 week under 0.15 bar (as in Figure 2c). Surprisingly, as the temperature ramp proceeds, an additional blueshift in the WGM pattern is produced, as shown in Figure 4a for a representative spectral region. Upon cooling back to 20 °C, the peaks did not return to their original positions, as evidenced in Figure 4b where the spectral shift of the mode highlighted in Figure 4a is followed as a function of temperature, revealing an irreversible shift. This behavior is consistent with the residual evaporation of isopropanol trapped within the PSμP, indicating that low-pressure storage at room temperature was insufficient to achieve complete solvent removal. A second heating ramp up to 80 °C caused a further blue-shift, with a total displacement of Δλ = −1.2 nm.

**Figure 4:**
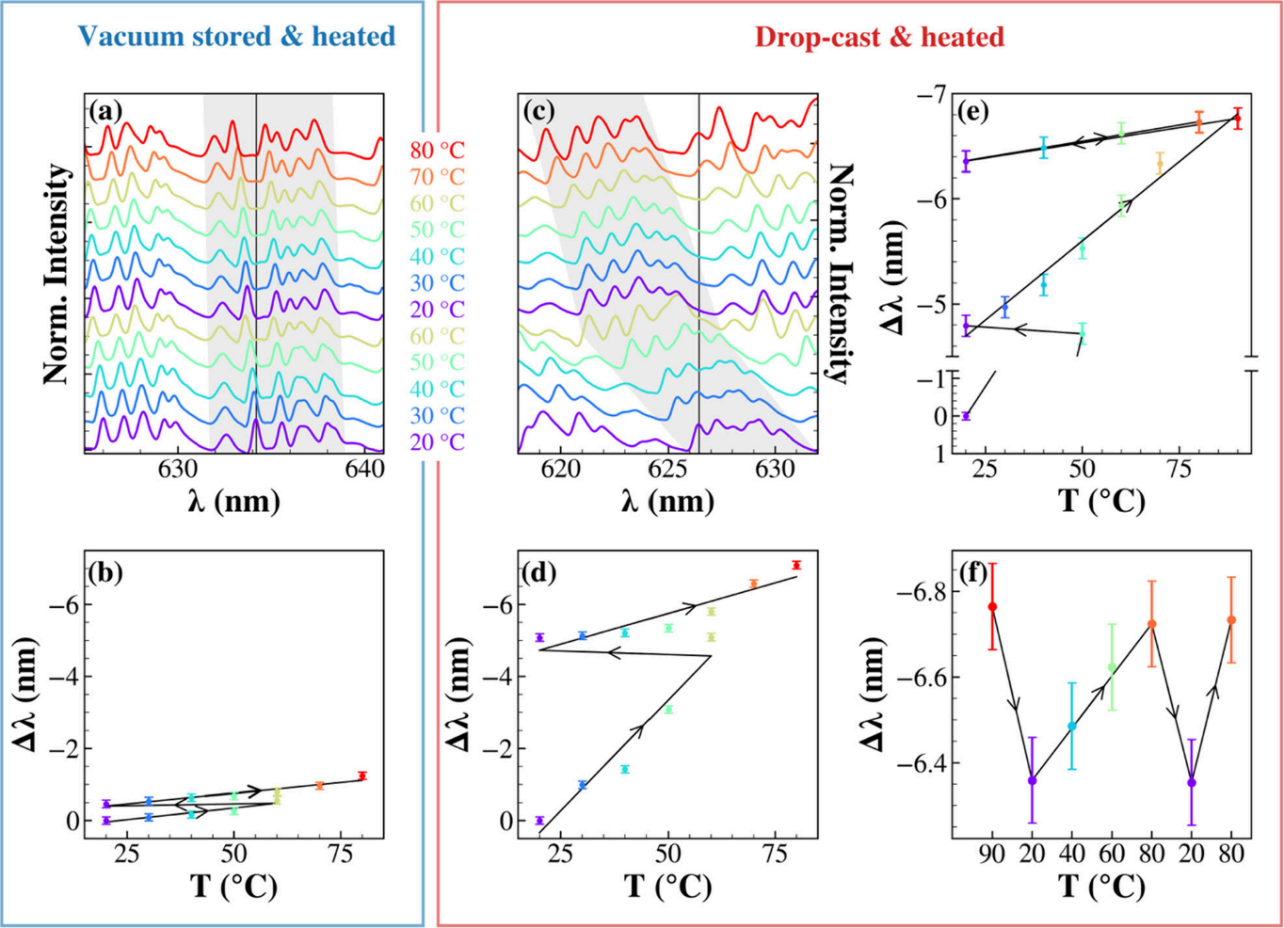
Thermal response of WGM peaks in an individual QD-PSμPs under two conditions: after 1 week of mild vacuum storage (blue left box) and immediately after drop-casting (red right box). Panels (a) and (c) display the evolution of a set of WGM peaks with temperature (20–80 °C) in the two cases. Panels b and d summarize the corresponding spectral shift (Δλ) of a representative WGM peak, highlighted by the vertical lines in panels (a) and (c). Arrows indicate temperature sweep direction. (e) Δλ during repeated thermal cycles. (f) Reversibility of Δλ after thermal stabilization.

In a second experiment, we applied the same thermal protocol to freshly drop-cast QD-PSμP. The corresponding temperature evolution of the spectrum is shown in Figure 4c. In this case, the first heating ramp from 20 to 60 °C induced a more pronounced blueshift of Δλ ∼ −5 nm, again irreversible upon cooling (Figure 4d). Heating to 80 °C resulted in a cumulative total blueshift of 7.2 nm relative to the initial spectrum, as better shown in Figure 4e for a representative peak. This larger displacement is consistent with a higher initial solvent content. Nevertheless, after cooling at 20 °C and repeating incremental heating steps of 10 °C, a progressive blueshift was still observed. We then performed a heating ramp to 90 °C to exceed the boiling point of isopropanol (82.6 °C at atmospheric pressure).^37^ Interestingly, beyond this point, thermal cycling produced fully reversible spectral shifts, as shown in Figure 4e and highlighted in Figure 4f, indicating complete solvent removal.

Once all isopropanol is removed, irreversible shifts disappear, and the remaining small, reversible response can be attributed to the intrinsic thermoelastic properties of the polymeric microresonator. By differentiating eq 1 and truncating at the first order of approximation, the temperature dependence of the WGM wavelength can be expressed as

2 $$\frac{1}{\lambda }\frac{d\lambda }{dT}=\alpha +\frac{1}{n}\frac{dn}{dT}$$

where α and *dn*/*dT* are the thermal expansion and thermo-optic coefficients of the microresonator, respectively. When both have the same sign, their contributions increase, resulting in monotonic spectral shifts. For example, silica possesses α≈10^–6^ K^–1^ and *dn*/*dT*≈10^–6^ K^–1^,^38^ leading to dominant redshifts upon heating. In contrast, polystyrene has a positive α ≈ 7 × 10^–5^ K^–1^ but a negative d*n*/d*T* ≈ −1.2 × 10^–4^ K^–1^,^38^ so that the two contributions in eq 2 nearly cancel each other, resulting in a very small net temperature dependence. This is consistent with the measured value of Δλ/Δ*T* ∼ −6 × 10^–3^nm/°C as calculated from data reported in Figure 4f, and confirms that once thermally stabilized, QD-PSμPs display relatively low thermal sensitivity. Therefore, QD-PSμP displays temporal (Figure 1) and thermal spectral drifts (Figure 4) which cannot be eliminated by a prolonged exposure to mild vacuum (Figure 2). However, the adopted thermal treatment does suggest an effective activation route for otherwise unstable photonic microlabels, yielding temperature- and time-invariant spectral fingerprints, appealing characteristics for robust photonic labeling applications.

Based on these results, we propose a thermal stabilization protocol where a freshly drop-cast QD-PSμP is heated at 85 °C for 1 h. Figure 5 compares the μ-PL spectrum of the particle before treatment (blue-filled) and immediately after heating (black line), with colored arcs marking groups of peaks within the dense WGM pattern. Remarkably, when measuring the spectrum of the same particle after 60 days in ambient conditions (gray line), we observed outstanding spectral repeatability, maintaining an identical WGM progression, and preserved relative peak intensities. From a technological standpoint, this simple thermal treatment not only stabilizes the WGM spectral fingerprints, thus enabling reliable photonic labeling, but also establishes hybrid QD-PSμPS as versatile, ultrasensitive probes for monitoring solvent release and nanoscale environmental changes.

**Figure 5:**
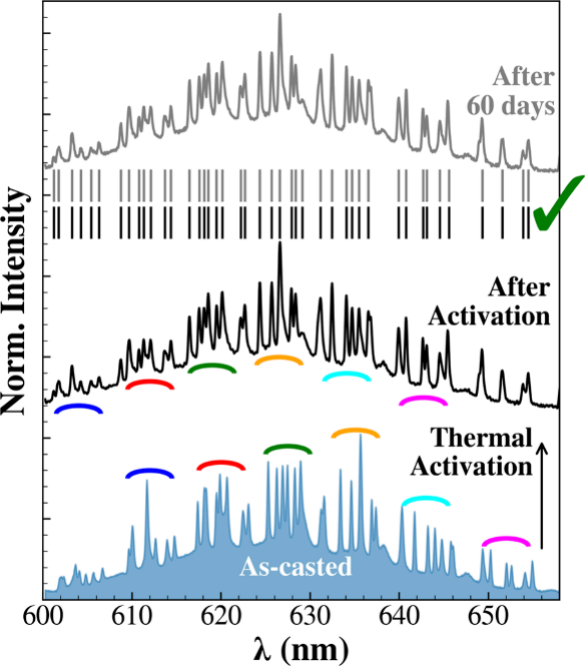
μ-PL spectrum of a QD-PSμP recorded a few minutes after drop-casting (blue-filled) and after thermal activation (black line), showing a distinct blueshift of the main WGM groups (highlighted by colored arcs). The gray spectrum corresponds to the same measured 60 days later, after storage under ambient conditions. The spectral positions of WGMs remain unchanged after thermal activation, enabling their use as a stable photonic barcode.

In summary, our study of WGMs in hybrid QD-PSμP microcavities shows that the unique spectral fingerprints of individual particles encode a highly sensitive record of their local environment. Temporal tracking of WGMs reveals that residual solvent drives irreversible blueshifts, while a simple thermal protocol suppresses these drifts, yielding long-term stable and reproducible WGM patterns. This thermal treatment is material-intrinsic and irreversible and requires no additional fabrication steps. After the stabilization protocol, the QD-PSμP resonators exhibit low thermal sensitivity due to near-compensation between the positive thermal expansion and the negative thermo-optic coefficient of polystyrene. Beyond secure photonic labeling, this approach establishes WGM spectroscopy as a noninvasive, real-time probe of structural and compositional dynamics in soft photonic architectures. More broadly, our method offers a general framework for designing responsive photonic materials, controlled-release platforms, and dynamically reconfigurable microresonators.

## Experimental Methods

### QD Synthesis

CdSe/CdS QDs were synthesized by following the literature^29^ with modifications reported in our previous publication.^39^

### Assembly of QD-PSμPs

Polystyrene microparticles (PSμPs, nominal diameter 10 μm) purchased from Sigma-Aldrich were loaded with synthesized QDs using a solvent-assisted procedure adapted from literature protocols.^18^

PSμPs were first dispersed in 450 μL of isopropanol (IPA) at a concentration of 9 × 10^6^ particles/mL in a glass vial and kept under continuous magnetic stirring. A total of 50 μL of a 1.6 μM­(dot) solution of QDs in chloroform were added dropwise to PSμP suspension. Upon addition, chloroform is expected to partially penetrate the polystyrene matrix, inducing a transient swelling that generates nanoscale diffusion pathways within the polymer. These pathways facilitate the migration of QDs from the mixed solvent phase into the polymer network. Simultaneously, the limited miscibility of chloroform with isopropanol creates local composition gradients and modifies the solvation environment of the QD ligands. These effects contribute to a mild colloidal destabilization of the QDs, promoting their adsorption onto the PSμP surface and subsequent incorporation into the swollen polymer shell. The mixture was stirred for 12 h at room temperature. QD-PSμPs were then separated from unbound QDs by centrifugation at 6000 rpm for 5 min. The supernatant containing excess QDs was discarded, while the pellet was redispersed in fresh isopropanol and stored at 4 °C.

### UV–Vis Measurements

UV–vis absorption spectrum of diluted colloidal QD dispersion in chloroform was recorded using an Avantes fiber-optic spectrophotometer equipped with a multichannel CMOS detector, providing 1 nm spectral resolution. A combined deuterium/halogen lamp was used as a broadband illumination source.

### Photoluminescence Measurements

Photoluminescence (PL) spectrum of a diluted colloidal dispersion of QDs in chloroform was obtained by using a spectrophotometer (Acton SpectraPro 2300i monochromator coupled to a Princeton Instruments P400 CCD camera) under excitation from a 532 nm CW laser diode provided by Thorlabs. A 300 grooves/mm grating was used for the measurements, allowing for a spectral resolution of 1 nm.

Absolute quantum yield (QY) measurements of a diluted colloidal dispersion of QDs were obtained using an integration sphere (Labsphere) under 532 nm excitation provided by a diode laser (Thorlabs). The signal was detected on a fiber-optic spectrophotometer (Avantes) equipped with a multichannel CMOS detector. The QY value was estimated within a 5% uncertainty.

### Microphotoluminescence Measurements

μPL measurements were carried out using a LabRam HR-Evolution Spectrometer system (HORIBA France SAS, Lyon, France) coupled to a confocal microscope. A pinhole aperture equal to 200 μm and a 50× LWD objective were used throughout the measurements. A laser excitation source (532 nm wavelength) was used to record all spectra. Laser power was reduced to 1 mW by using a ND filter to avoid damaging the samples with the intense focused light. The spectrometer was equipped with a 600 lines/mm grating, which provides a resolution better than 0.2 nm.

All temperature-dependent measurements were carried out by placing the samples into a THMS600-PS cell (Linkam Scientific Instruments, Salfords, United Kingdom). All temperature ramps were performed in an ambient atmosphere with an open cell lid. The set temperature was reached at a rate of 100 °C/min.

## Supplementary Material



## Notes

Theoretical modeling details, additional supporting data, and supplementary figures (PDF)
