# Supplementary material for: A Versatile Approach to Stabilize Whispering Gallery Microresonators Toward Reliable Photonic Labeling
Source: J Phys Chem Lett. 2026 Jan 29;17(7):2042–8. doi: 10.1021/acs.jpclett.5c03840 (PMC12927022; doi:10.1021/acs.jpclett.5c03840)
Supplement: Supplementary file 1 [file jz5c03840_si_001.pdf]

Supporting Information

A Versatile Approach to Stabilize Whispering Gallery Microresonators Towards Reliable Photonic Labeling

M. Reale[1,2], A. Madonia[1], S. Agnello[1,3], M. Cannas[1,2], E. Marino[1], A. Sciortino[1,3], F. Messina[1,3]

[1]Department of Physics and Chemistry E. Segrè, University of Palermo, via Archirafi 36, 90123, Palermo (Italy)

[2]Consorzio Interuniversitario Nazionale per la Scienza e Tecnologia dei Materiali (INSTM), via G. Giusti 9, 50121, Firenze (Italy)

[3]ATEN Center, University of Palermo, Viale delle Scienze Ed. 18, I-90128 Palermo, (Italy)

1 Theoretical modeling – WGM Evolution During Solvent Evaporation

The evolution of the whispering gallery mode (WGM) resonances observed during the evaporation of isopropanol from the QD-loaded polystyrene microparticles (QD-PSμP) can be modeled as a coupled optical–structural response occurring in two sequential stages.

Stage 1: External solvent evaporation and increase of refractive index contrast

Immediately after drop-casting the particle suspension, each microparticle is surrounded by a relatively thick layer of isopropanol. At this stage, the refractive index of the surrounding medium can be approximated as that of pure isopropanol: $n_{env} = n_{IPA}$. As the isopropanol layer evaporates, n_{env} progressively decreases and approaches the refractive index of air: $n_{AIR} \approx 1$ (Figure 3a). This increasing refractive-index enhances the contrast between the microparticle and its environment, enhancing the optical confinement within the cavity, resulting in a higher Q-factor and sharper WGM resonances. Meanwhile, the microparticle retains a fraction of isopropanol trapped within the polymer matrix. This internal solvent volume fraction is denoted ϕ_0^{IPA}, while the initial QD volume fraction is denoted ϕ_0^{QD}, and both can be treated as parameters.

To estimate the effective refractive index of the hybrid particle, we employ the Maxwell-Garnett effective medium approximation,[1] considering QDs and isopropanol guest inclusions embedded in a polystyrene matrix:

$$n_{eff,0}(\phi_0^{IPA}, \phi_0^{QD}) = \sqrt{n_{PS}^2 \frac{1 + 2\beta_{IPA}\phi_0^{IPA} + 2\beta_{QD}\phi_0^{QD}}{1 - \beta_{IPA}\phi_0^{IPA} - \beta_{QD}\phi_0^{QD}}}$$

with

$$\beta_{IPA} = \frac{n_{IPA}^2 - n_{PS}^2}{n_{IPA}^2 + 2n_{PS}^2}$$

$$\beta_{QD} = \frac{n_{QD}^2 - n_{PS}^2}{n_{QD}^2 + 2n_{PS}^2}$$

The effective refractive index therefore depends parametrically on ϕ_0^{IPA} and ϕ_0^{QD}. Considering a particle with initial radius R_0, and focusing on a WGM denoted as λ, with radial mode number i and

angular mode number l, the spectral position of the resonance can be evaluated, using eq. (1) from the main text, as:

$$\lambda_0(\phi_0^{IPA}, \phi_0^{QD}) = \frac{2\pi R_0 n_{eff,0}(\phi_0^{IPA}, \phi_0^{QD})}{Q\left(\frac{1}{2} + l, i; n_{eff,0}(\phi_0^{IPA}, \phi_0^{QD}), n_{IPA}\right)}$$

At the end of this first stage the external medium refractive index transition from n_{IPA} to $n_{AIR} \approx 1$, and resonance is expected to shift to:

$$\lambda_1(\phi_0^{IPA}, \phi_0^{QD}) = \frac{2\pi R_0 n_{eff,0}(\phi_0^{IPA}, \phi_0^{QD})}{Q\left(\frac{1}{2} + l, i; n_{eff,0}(\phi_0^{IPA}, \phi_0^{QD}), 1\right)}$$

We can thus quantify the spectral shift of the mode occurring during this first stage, as

$$\Delta\lambda_{(i)}(\phi_0^{IPA}, \phi_0^{QD}) = \lambda_1(\phi_0^{IPA}, \phi_0^{QD}) - \lambda_0(\phi_0^{IPA}, \phi_0^{QD}).$$

Its dependence on $\phi_0^{IPA}, \phi_0^{QD}$ is reported in the 3D map of Figure 3b, assuming initial radius $R_0 = 5.1$ µm, focusing on a WGM of radial mode number $i = 1$, and a representative angular mode number $l = 71$ (selected because lying within the spectral region of interest).

Stage 2: Internal solvent loss and structural shrinkage

Once the external isopropanol layer has fully evaporated, a residual fraction of solvent remains inside the polymer network of the microparticle. This internal isopropanol gradually desorbs, reducing the isopropanol volume fraction from its initial value ϕ_0^{IPA} down to $\phi^{IPA} = 0$. This removal of solvent from within the matrix induces a partial densification and collapse of the polymer structure, leading to a gradual decrease of the particle radius from its initial value R_0 to a smaller final value R (Figure 3c). Because the total volume occupied by the QDs and the polystyrene are conserved during the process, the total volume reduction of the particle is equal to the initial amount of isopropanol present inside the matrix. This allows us to write:

$$V - V_0 = -V_0\phi_0^{IPA}$$

From this expression, it follows that after complete evaporation of the internal isopropanol, the particle radius becomes:

$$R = R_0(1 - \phi_0^{IPA})^{1/3}$$

At the same time, the volume fraction of QDs increases due to the reduction in particle volume. The new QD volume fraction is therefore:

$$\phi^{QD} = \frac{\phi_0^{QD} V_0}{V} = \frac{\phi_0^{QD}}{1 - \phi_0^{IPA}}$$

The effective refractive index of the hybrid particle during this second stage can thus be calculated using again the Maxwell-Garnett effective medium approximation, considering the QDs as only guest inclusions embedded in a polystyrene matrix:

$$n_{eff} = \sqrt{n_{PS}^2 \frac{1 + 2\beta_{QD}\phi^{QD}}{1 - \beta_{QD}\phi^{QD}}} = \sqrt{n_{PS}^2 \frac{1 + 2\beta_{QD}\left(\frac{\phi_0^{QD}}{1 - \phi_0^{IPA}}\right)}{1 - \beta_{QD}\left(\frac{\phi_0^{QD}}{1 - \phi_0^{IPA}}\right)}}$$

This refractive index therefore depends parametrically on the initial volume fractions ϕ_0^{IPA} and ϕ_0^{QD} and so does the final spectral position of the WGM resonance after shrinkage:

$$\lambda_2(\phi_0^{IPA}, \phi_0^{QD}) = \frac{2\pi R_0(1 - \phi_0^{IPA})^{1/3} n_{eff}(\phi_0^{IPA}, \phi_0^{QD})}{Q\left(\frac{1}{2} + l, i; n_{eff}(\phi_0^{IPA}, \phi_0^{QD}), 1\right)}$$

The expected spectral shift associated with this second stage of internal solvent desorption can therefore be written as:

$$\Delta\lambda_{(ii)}(\phi_0^{IPA}, \phi_0^{QD}) = \lambda_2(\phi_0^{IPA}, \phi_0^{QD}) - \lambda_1(\phi_0^{IPA}, \phi_0^{QD}).$$

The dependence of $\Delta\lambda_{(ii)}$ on the initial parameters $\phi_0^{IPA}, \phi_0^{QD}$, assuming initial radius $R_0 = 5.1$ µm, focusing on a WGM of radial mode number $i = 1$, and a representative angular mode number $l = 71$, is reported in the 3D map of Figure 3d.

2 Supplementary Figures

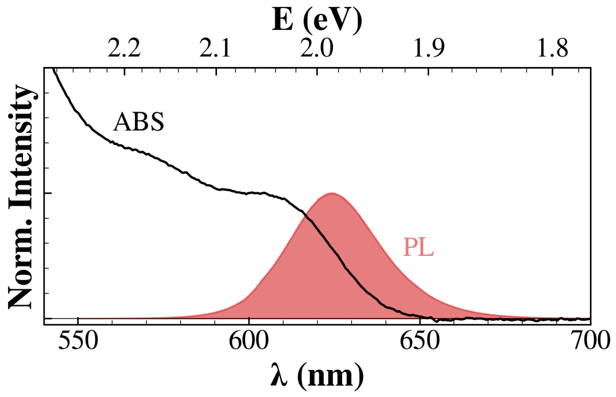

Figure S1: Absorption (ABS) and photoluminescence (PL) spectra of a QD dispersion in chloroform.

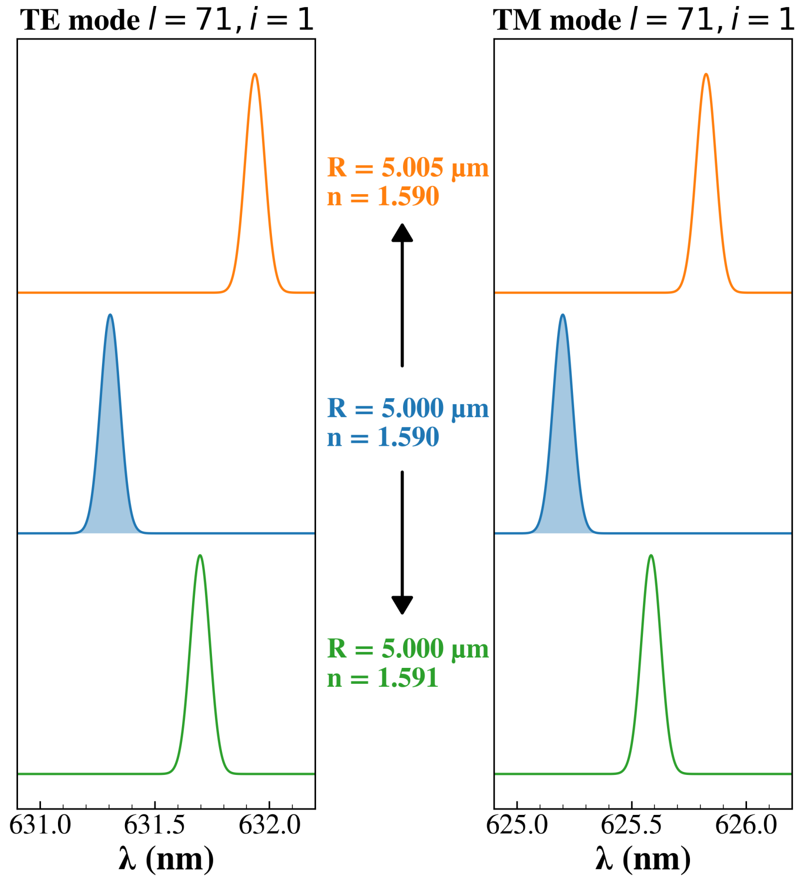

Figure S2: Effect of radius and refractive index changes on the spectral position of a representative WGM (l=71, i=1), calculated for both transverse electric (TE, on the left) and transverse magnetic (TM, on the right) polarization.

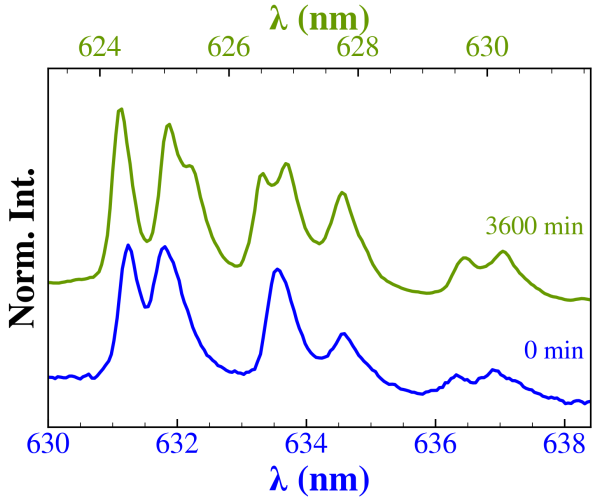

Figure S3: Zoomed-in view of a selected spectral region of the μ-PL spectrum of a QD-PSμP before (blue) and after (green) low pressure storage (0.15 bar), extracted from the data shown in Figure 2. The corresponding x-axis scales are shown in the same colors.

(1) Markel, V. A. Introduction to the Maxwell Garnett Approximation: Tutorial. *Journal of the Optical Society of America A* **2016**, *33* (7), 1244. https://doi.org/10.1364/JOSAA.33.001244.
